# Coinfection modulates inflammatory responses, clinical outcome and pathogen load of H1N1 swine influenza virus and *Haemophilus parasuis* infections in pigs

**DOI:** 10.1186/s12917-017-1298-7

**Published:** 2017-12-04

**Authors:** Małgorzata Pomorska-Mól, Arkadiusz Dors, Krzysztof Kwit, Ewelina Czyżewska-Dors, Zygmunt Pejsak

**Affiliations:** Department of Swine Diseases, National Veterinary Research Institute, Partyzantów 57, 24-100 Pulawy, Poland

**Keywords:** Pigs, Respiratory co-infections, Disease severity, Immunity, Pathogens shedding

## Abstract

**Background:**

Respiratory co-infections are important factor affecting the profitability of pigs production. Swine influenza virus (SIV) may predispose to secondary infection. *Haemophilus parasuis* (*Hps*) can be a primary pathogen or be associated with other pathogens such as SIV. To date, little is known about the effect of coinfection with SIV and *Hps* on the disease severity and inflammatory response and the role of *Hps* in the induction of pneumonia in the absence of other respiratory pathogens. In the study we investigated the influence of SIV and *Hps* coinfection on clinical course, inflammatory response, pathogens shedding and load at various time points following intranasal inoculation. The correlation between local concentration of cytokines and severity of disease as well as serum acute phase proteins (APP) concentration has been also studied.

**Results:**

All co-infected pigs had fever, while in single inoculated pigs fever was observed only in part of animals. Necropsy revealed lesions in the lungs all SIV-inoculated and co-inoculated pigs, while in *Hps*-single inoculated animals only 1 out of 11 pigs revealed gross lung lesions. The SIV shedding was the highest in co-inoculated pigs. There were no differences between *Hps*-single inoculated and co-inoculated groups with regard to *Hps* shedding. The significant increase in *Hps* titre in the lung has been found only in co-inoculated group. All APP increased after co-infection. In single-inoculated animals various kinetics of APP response has been observed. The lung concentrations of cytokines were induced mostly in SIV + *Hps* pigs in the apical and middle lobe. These results correlated well with localization of gross lung lesions.

**Conclusions:**

The results revealed that SIV increased the severity of lung lesions and facilitated *Hps* (PIWetHps192/2015) replication in the porcine lung. Furthermore, *Hps* influenced the SIV nasal shedding. Enhanced *Hps* and SIV replication, together with stronger systemic and local inflammatory response contributed to a more severe clinical signs and stronger, earlier immune response in co-inoculated animals. We confirmed the previous evidence that single-*Hps* infection does not produce significant pneumonic lesions but it should be in mind that other strains of *Hps* may produce lesions different from that reported in the present study.

## Background

Respiratory infections in pigs are very important factor affecting the profitability of pig production [1, 2]. Although various bacteria or viruses could induce the respiratory infection separately, it has commonly been caused by coinfection with more pathogens under field conditions [1–3]. The most important infectious agents responsible for infection of the respiratory tract in pigs are: swine influenza virus (SIV), porcine reproductive and respiratory syndrome virus (PRRSV), *Pasteurella multocida* (Pm), *Actinobacillus pleuropneumoniae* and *Mycoplasma hyopneumoniae* [2, 4–6]. Besides, the above mentioned pathogens, the *Haemophilus parasuis (Hps)* can also be recovered from the lungs of pigs with pneumonia [1, 7–10]. In these cases *Hps* is often isolated along with other bacterial or viral pathogens and, therefore, the role of *Hps* in producing pneumonia is not clear [8, 11].

Bacterial pneumonia secondary to influenza is often observed in pigs [12]. SIV is a significant contributor to the respiratory diseases and may predispose to secondary bacterial infection. *Hps* is an important and common respiratory pathogen of pigs [13]. It can be a primary pathogen or be associated with other diseases such as SIV [3, 8]. It could be also isolated from nasal cavity, tonsils and trachea of apparently healthy pigs [8, 14]. Under favorable conditions, *Hps* can cause severe systemic infection characterized by fibrinous polyserositis, arthritis and meningitis [8, 11, 14]. Factors leading to systemic infection by *Hps* have not been clarified to date [9, 14].

Although there are previous reports of experimental reproduction of *Hps* or SIV infection in conventional pigs, little is known about the effect of concurrent infection with SIV and *Hps* on the disease severity and inflammatory response in pigs, even if this coinfection is common under field conditions [13, 15–17]. There are also limited data on the role of *Hps* in the production of pneumonia in the absence of other respiratory pathogens. Furthermore, the kinetics of acute phase protein (APP) response in SIV/*Hps* co-infected pigs has not been studied to date. As it has been shown for other pathogens, the exposure to several pathogens can lead to a stronger APP response, as compare to single infection [18–20]. Thus, in order to investigate the influence of SIV and *Hps* coinfection on clinical outcome, both local and systemic inflammatory response as well as pathogen shedding and load at various time points following intranasal inoculation, three experimental infections (*Hps*- and SIV-single infection, SIV/*Hps* co-infection) has been performed in the present study. The correlation between local concentration of cytokines and severity of infection (clinical score, lung score) as well as serum APP concentration has been also studied.

## Results

### Clinical signs

**Fig. 1:**
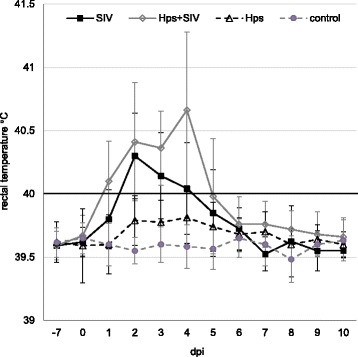
Rectal temperature (mean ±SD) in pigs single or dual inoculated with swine influenza virus (SIV) and/or *Haemophilus parasius* (Hps). Number of pigs affected: *Hps* + SIV 11/11; SIV 7/11; *Hps* 3/11

**Fig. 2:**
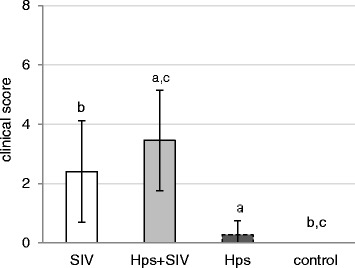
Clinical score (mean±SD) of pigs single or dual inoculated with swine influenza virus (SIV) and/or *Haemophilus parasius* (Hps). The significant differences between groups are indicated with the same superscripts

All pigs from co-infected group had fever for at least one day (rectal temperature ≥40° C). In SIV – inoculated group fever was observed in 7 out of 11 pigs, while in *Hps* - inoculated pigs only in 3 out of 11 pigs (Fig. 1). The mean clinical scores (±SD) in all groups are presented in Fig. 2. In single-inoculated pigs, the individual clinical score ranged from 0 to 1 (*Hps*) or from 0 to 5 (SIV), while in co-inoculated pigs the individual clinical score ranged from 1 to 6. Pigs from the control group did not revealed clinical signs of any disease. Significant differences were observed between mean clinical score in SIV- and *Hps* + SIV – inoculated pigs and the controls (*p*≤0.05). There was also significant difference between co-inoculated and *Hps*-inoculated group (*p*≤0.05). No differences were observed between mean clinical score in SIV-single inoculated and co-inoculated animals as well as between *Hps*-inoculated and control animals.

### Pathological examination

**Fig. 3:**
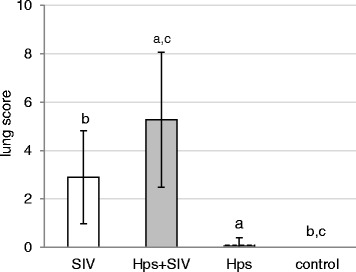
Lung score (mean±SD) of pigs single or dual inoculated with swine influenza virus (SIV) and/or *Haemophilus parasius* (Hps). The significant differences between groups are indicated with the same superscripts

Postmortem examination revealed macroscopic lesions (well-demarcated plum-colored or dark red lesions) in the lungs all SIV-inoculated and co-inoculated pigs, while in *Hps*-single inoculated animals only 1 out of 11 pigs revealed macroscopic changes in the lung. None of the control pigs had visible pneumonia. The mean lung scores (±SD) are presented in Fig. 3. There were no significant differences between lung score observed at different days post inoculation within each group (*p* > 0.05). Significant differences were found between mean lung score observed co-inoculated and *Hps*-inoculated groups and control animals (*p* < 0.05). There was no difference between lung score noted in co-inoculated and SIV-only inoculated animal. No differences were also found between mean lung score in *Hps*-inoculated and control pigs.

### Pathogens shedding and load

**Fig. 4:**
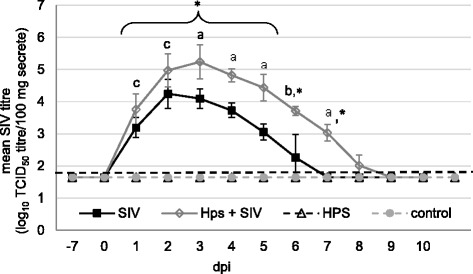
Nasal virus shedding (mean ±SD) after inoculation of pigs with swine influenza virus (A/Poland/Swine/14131/2014) and or *Haemophilus parasius*. Mean virus titres (determined by cell culture) in nasal swabs collected during study period. The dashed line represents the detection limit. * - the significant differences compared to control group; a - the significant differences between inoculated groups; b - the significant differences between SIV and *Hps* + SIV inoculated groups; c – the significant differences between SIV, *Hps* + SIV and *Hps*

**Fig. 5:**
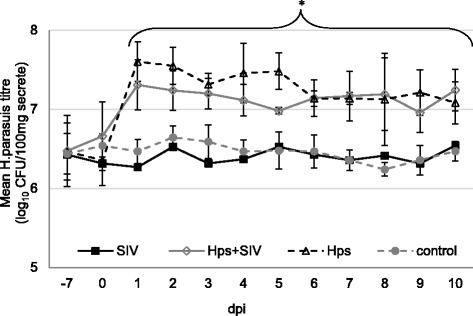
Nasal bacteria shedding (mean ±SD) after inoculation of pigs with *Haemophilus parasius*. Mean colony forming units (CFU) (determined by quantitative PCR) in nasal swabs collected during study period. * - the significant differences compared to control and SIV group

The highest SIV shedding (expressed as mean SIV titre (log_10_ TCID_50_ titre/100 mg secrete from nasal swabs) was observed in co-inoculated pigs. Significant differences between single-SIV and dual inoculated pigs were found from 3 to 7 DPI (*p* < 0.05). Compared to control pigs, significantly higher (*p* < 0.05) mean SIV titers were observed in both SIV-single inoculated pigs and co-inoculated pigs from 1 to 5 DPI and 1 to 7 DPI, respectively (Fig. 4.). The mean *Hps* titre in the nasal swabs (expressed as log_10_ CFU titre/100 mg secretion) in piglets from SIV + *Hps* and *Hps* groups increased significantly from day 1 post inoculation and remaining at the significantly higher level to the end of study (*p* < 0.05). There were no differences between *Hps*-single inoculated and co-inoculated groups with regard to *Hps* nasal shedding (Fig. 5.).

**Fig. 6:**
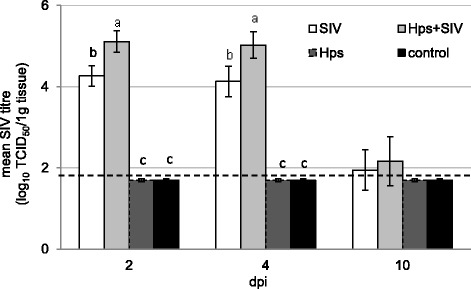
Swine influenza virus titre (mean ±SD) (determined by cell culture) in the lung at 2, 4 and 10 days after single or dual inoculation of pigs with swine influenza virus and/or *Haemophilus parasius*. The dashed line represents the detection limit. Columns with various superscripts within the same day of study differ significantly

**Fig. 7:**
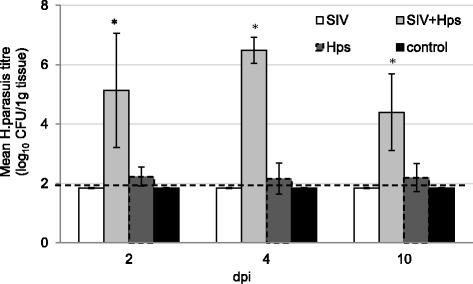
*H. parasuis* titres (mean ±SD) (determined by quantitative PCR) in the lung at 2, 4 and 10 days after single or dual inoculation of pigs with swine influenza virus and/or *Haemophilus parasius*. The dashed line represents the detection limit. * - significant differences compared to remaining groups

The SIV and *Hps* titres in the lungs at 2, 4 and 10 dpi are presented in Figs. 6 and 7, respectively. All pigs inoculated with SIV were virus positive in samples taken from right lungs at 2 and 4 DPI. At 10 DPI the virus was detected in 1 out of 5 lungs in SIV-single inoculated animals, while in pigs co-inoculated with *Hps* in 2 out of 5 lungs and no significant differences between virus inoculated groups were found. The mean SIV TCID_50_ titres were highest in co-inoculated pigs at 2 and 4 DPI (*p* < 0.05).

The significant increase in *Hps* titre in the lung were found only in co-inoculated group (*p* < 0.05). The significant differences were detected between the mean titre of *H. parasuis* in the lungs taken from pigs from *Hps* and SIV + *Hps* groups during whole study period (2, 4 and 10 DPI) (*p* < 0.05).

### Humoral immune response to SIV and *Hps*

**Fig. 8:**
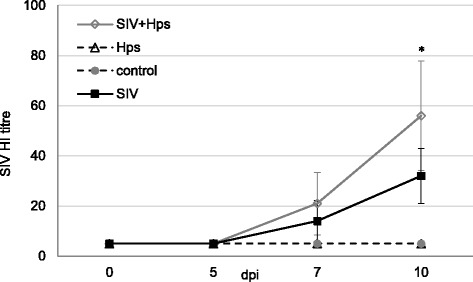
Mean (±SD) HI antibody titres against A/Poland/Swine/14131/2014 SIV in of pigs single or dual inoculated with swine influenza virus (SIV) and/or *Haemophilus parasius* (Hps). * - significant differences compared to groups not inoculated with SIV

**Fig. 9:**
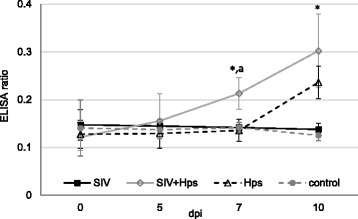
Mean (±SD) level of antibodies against *Haemophilus parasius* in of pigs single or dual inoculated with swine influenza virus (SIV) and/or *Haemophilus parasius* (*Hps*) * - significant differences compared to control group; a - significant differences between co-inoculated pigs and *Hps* single-inoculated groups. ELISA ratio (ER) was calculated as directed by the manufacturer (Swinecheck®HPS, Biovet, Canada) according to the following formula: $\mathrm{Er}=\frac{\mathrm{OD}\ \mathrm{of}\  \mathrm{test}\  \mathrm{serum}\  \mathrm{from}\  \mathrm{wells}\  \mathrm{with}\ \mathrm{Ag}\hbox{-} \mathrm{OD}\ \mathrm{of}\  \mathrm{test}\  \mathrm{serum}\  \mathrm{from}\  \mathrm{wells}\  \mathrm{with}\mathrm{out}\ \mathrm{Ag}}{\mathrm{mean}\ \mathrm{OD}\ \left(+\right)\ \mathrm{from}\  \mathrm{wells}\  \mathrm{with}\ \mathrm{Ag}\hbox{-} \mathrm{mean}\ \mathrm{OD}\ \left(+\right)\ \mathrm{from}\  \mathrm{wells}\  \mathrm{with}\mathrm{out}\ \mathrm{Ag}\ }$ (+) - positive control, Ag - antigen

The onset of specific humoral response after inoculations with SIV and/or *Hps* is presented in Figs. 8 and 9, respectively.

A specific humoral response against SIV was observed at 7 DPI in 80% of pigs (4/5) co-inoculated and 40% of pigs (2/5) single inoculated with SIV. All animals inoculated with SIV (single or dual) developed specific antibodies at 10 DPI (the HI titre ranged from 20 to 80 in co-inoculated pigs and from 20 to 40 in SIV-only inoculated group). There were no differences with regard to HI titre at 7 and 10 DPI between groups inoculated with SIV (single or dual) (*p*≥0.05). None of pigs not inoculated with SIV had antibodies against this pathogen (<20 HI titre).

Significant differences between co-inoculated and single-inoculated or control pigs with regard to *Hps* specific antibodies was observed at 7 DPI (*p*≤0.05). Furthermore, in groups inoculated with *Hps* (single or dual) the mean ELISA ratio at 10 DPI was significantly higher than in SIV – inoculated and control animals (*p*≤0.05). The ELISA ratio in control group did not differ significantly from the observed in SIV-inoculated pigs during study period.

### Systemic levels of acute phase proteins

**Fig. 10:**
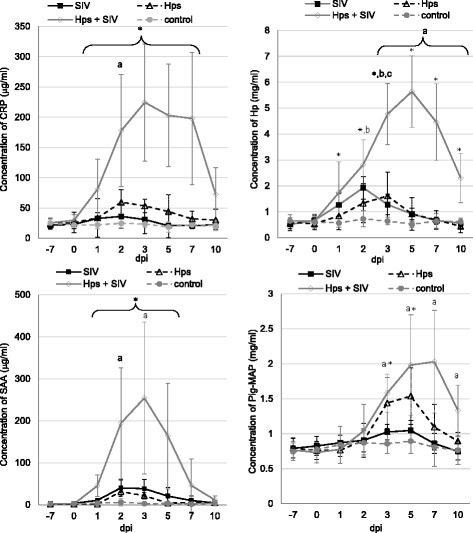
Concentrations of SAA, Pig-MAP, Hp and CRP (mean ±SD) in serum of pigs before and on various time-point after single or dual inoculation with swine influenza virus (SIV) and/or *Haemophilus parasius* (Hps). CRP: * - significant differences between co-inoculated and control group; a - significant differences between *Hps*-inoculated and control group; Hp: a- significant differences between single and dual-inoculated groups; * - significant differences between co-inoculated and control pigs; b - significant differences between SIV and control groups, c - significant differences between *Hps* and control groups. SAA: * - significantly differences between co-inoculated and control pigs, a – significant differences between single-inoculated and control pigs. Pig-MAP: a – significant differences between co-inoculated and control pigs; * - significant differences between Hps and control group

All studied APP increased after co-infection, with mean maximum concentrations from 3 to 7 DPI (Fig. 10.) (*p* < 0.05). In single-inoculated animals different kinetics of acute phase response has been noted. In the control group concentrations of all APP were constant.

### C-reactive protein

Significant increase in the mean concentration of CRP compared to control pigs has been observed in co-inoculated animals from 1 to 7 DPI. At 2 DPI the significant difference compared to control group was noted also for the *Hps*-inoculated pigs (*p* < 0.05). The maximum mean level of CRP in co-inoculated animals was observed at 3 DPI and reached 224.61 ± 96.80 μg/ml (almost 8-fold increase comparing to the day 0 level). In pigs inoculated only with *Hps* the maximum mean concentration of CRP was observed at 2 DPI (2.5 fold increase comparing to day 0 level). In pigs from SIV group the mean levels of CRP did not differ significantly from that determined in the control animals.

### Haptoglobin

The concentration of Hp increased significantly in all inoculated groups comparing to control animals. The strongest and the most prolonged Hp response was found in SIV + *Hps* group. The mean concentration of Hp in this group had increased by 48 h after co-inoculation and were notably higher compared to control pigs (*p* < 0.05) to the end of study. Significant differences between single and dual-inoculated groups were observed from 3 to 10 DPI (*p* < 0.05). The highest mean concentrations of Hp in co-inoculated pigs were observed at 5 DPI. The mean maximal concentration was almost 9-fold higher, compared to the mean baseline concentration. The highest concentration of Hp in particular animal after coinfection reached 7.03 mg/ml (at 3 DPI). In pigs single inoculated with SIV or *Hps* only short-term increase in Hp level was recorded (at 2 and 3 DPI in SIV-inoculated and at 3 dpi in *Hps*-inoculated pigs).

### Serum amyloid

**A**. Significant increase of SAA concentration was found in all inoculated groups (*p* < 0.05). No significant changes were found in control pigs (*p*≥0.05). The strongest reaction has been noted in co-inoculated animals. The mean peak concnetration in mentioned group was detected at 3 DPI and reached 254.31 ± 181.32 μg/ml (over 100-fold increase comparing to the day 0 level). Significantly higher concentration compared to the controls was observed from 1 to 5 DPI (*p* < 0.05). In the remaining, single-inoculated groups the mean maximum concentrations were observed at 2 DPI (over 15-fold increase) and significant differences compared to controls were noted from 2 to 3 DPI (*p* < 0.05).

### Pig major acute phase protein

Baseline levels of Pig-MAP in experimental animals were found to be below 0.92 mg/ml. Concentration of Pig-MAP increased significantly 72 h after co-inoculation and inoculation with *Hps* (*p* < 0.05). In co-inoculated pigs the concentration of Pig-MAP remained significantly elevated till 10 DPI (end of study), while in *Hps*-single inoculated pigs till 5 dpi. The highest Pig-MAP mean levels were observed between 5 to 7 DPI. The maximum mean concentration of Pig-MAP, observed at 7 DPI in piglets from SIV + *Hps* group was almost 3 times higher compared to day 0-level (baseline concentration). There were no significant differences in the kinetics of Pig-MAP response between co-inoculated and *Hps*-single inoculated pigs. In pigs single inoculated with SIV as well as in control animals the level of Pig-MAP in serum was constant during study period.

### Cytokines – Local lung response

**Fig. 11:**
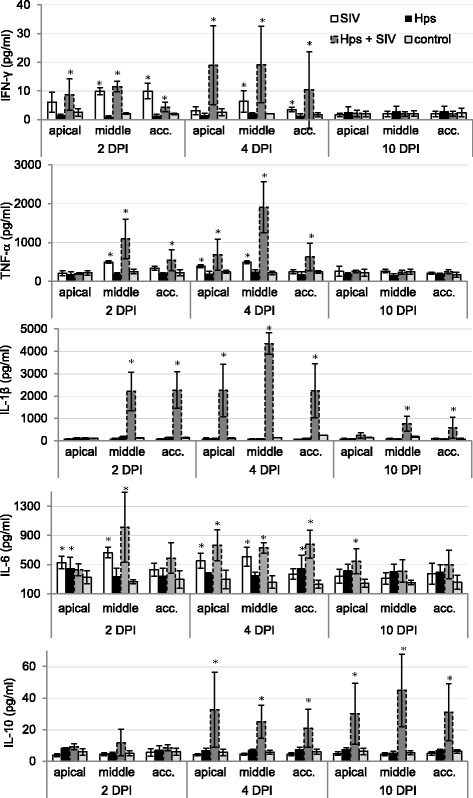
Quantification of cytokines in lung tissue of pigs single or dual inoculated with swine influenza virus (SIV) and/or *Haemophilus parasius* (Hps) (mean±SD). acc-accessory lobe; * - significant differences compared to control pigs (within the same day, at the same part of the lung)

In general the local concentrations of TNF-α, IFN-γ, IL-1β, IL-6 and IL-10 were induced mostly in SIV + *Hps* pigs (Fig. 11). In control pigs the concentrations of all investigated cytokines were relatively constant (*p*≥0.05).

At 2 DPI, concentrations of proinflammatory cytokines (IL-1β, IL-6, TNF-α) and IFN-γ were significantly higher in the lungs of co-inoculated pigs compared to controls (with exception of IL-1β and TNF-α in apical lobes). Furthermore significant differences were observed between co-inoculated and single inoculated pigs, with exception of IL-1β and TNF-α in apical lobes, and IFN-γ in middle and accessory lobes. In particular differences were noted between *Hps* and SIV + *Hps* group with regard to IFN-γ (all lobes) and TNF-α, IL-1β and IL-6 (except apical lobes) as well as between co-inoculated and SIV groups with regard to IFN-γ and IL-1β. No significant differences were found between control and single inoculated pigs with regard to IL-1β and IL-10 level in all lobes from the upper part of the right lung at that time. The highest concentrations of cytokines were generally observed in right middle lobes what positively correlated with the lesions detected during necropsy.

At 4 DPI the concentrations of all investigated cytokines were significantly higher in co-inoculated pigs, compared to controls (*p* < 0.05). No significant differences were observed with regard to IL-1β and IL-10 in single-inoculated groups, compared to controls (p≥0.05). With regard to SIV + *Hps* group the significantly higher concentration of investigated cytokines was observed in all evaluated parts of the lung. In SIV inoculated animals significantly higher concentration of IFN-γ compared to control group was observed in middle and accessory lobes, IL-6 in all lobes and TNF-α in apical and middle lobes. No significant increase in the level of cytokines tested was observed in *Hps* inoculated group (*p*≥0.05).

At 10 DPI the lung concentrations of cytokines generally decreased compared to 2 and 4 DPI, but in the case of IL-1β, IL-6 and IL-10 (in co-inoculated animals) were still significantly higher compared to controls (*p* < 0.05). Significantly greater concentrations were observed for IL-1β with regard to middle and accessory lobes, for IL-6 with regard to apical lobe and for IL-10 with regard to all lobes (*p* < 0.05). No significant differences between local concentrations of all investigated cytokines in single-inoculated and control animals were found for all lung lobes at 10 dpi.

Summarizing, the maximum concentrations of cytokines in general were observed at 4 DPI. The highest concentrations of most cytokines were found in the apical and middle lobe. These results correlated well with localization of gross lung lesions. Significant correlations were found between lung concentration of IL-1β, IL-6, IFN-γ and TNF-α and pathological changes in the lungs (R-Spearman = 0.65; 0.63; 0.71 and 0.62 respectively; *p* < 0.05). A positive correlation was also observed between concentrations of IL-1β and IFN-γ in the lungs and clinical signs (R-Spearman = 0.64 and 0. 67 respectively, p < 0.05). No significant correlation between local cytokine concentration and systemic APP response were found.

## Discussion

Swine influenza is commonly characterized by fever, respiratory and systemic nonspecific symptoms (i.e. loss of appetite, apatia) [16]. In the enzootic form, clinical signs may be less obvious. Subclinical infection is also quite common [17, 21, 22]. During uncomplicated infection the morbidity can be as high as 100% but the mortality is relatively low (ranges from less than 1% to 4%). The most common complications of swine influenza are secondary bacterial pneumonia and PRDC [3, 12]. Coinfections often lead to overproduction of cytokines that may be harmful to the host [12]. There are several mechanisms, by which SIV infection predisposes to secondary bacterial infection, including: increased expression of cell receptors leading to increased colonization and modification of host immune responses (i.e. impairing of phagocytic function of alveolar macrophages) [23–26]. Furthermore, it has been shown that damage caused by SIV in the respiratory tract (i.e. loss of cilia, extrusion of mucus, exudation, necrosis and metaplasia of airway epithelium), can reduce the ability of the host to clear the bacterial superinfection [2, 21]. *Hps* is one of the pathogens which may complicate swine influenza and be one of the etiological agents of PRDC [2, 3, 8, 27].

The mechanism by which SIV affects the host’s susceptibility as well as its immune response to secondary bacterial infections has not been fully elucidated. Previous research reviled that interactions among multiple pathogens can lead to an exacerbated inflammatory response and increased severity of infections [12, 28–30]. For example, gross lesions in the lungs and magnitude of APP response in pigs co-infected with SIV and *Pasteurella multocida* were more intensive compared to animals infected only with SIV or *Pasteurella multocida* [28]. Similar results were observed in pigs co-inoculated with SIV and *A. pleuropneumoniae* [19]. Co-infection with SIV and *A. pleuropneumoniae* potentiated the severity of lung lesions caused by SIV and enhanced virus replication in the lung and nasal SIV shedding. Enhanced SIV replication contributed to a more severe clinical course of the disease as well as earlier and higher magnitude immune and inflammatory responses [19]. Loving et al. [12] reported that SIV infection increased *Bordetella bronchiseptica* (Bb) colonization and increased the production of proinflammatory cytokines likely to exacerbate lung lesions. Pulmonary lesions in the co-infected pigs were more intense compared to SIV-only or Bb-only groups. The type I interferon, IL-1β and IL-8 were also significantly elevated in lungs of co-infected pigs.

There is limited data on the influence of SIV and *Hps* coinfection on the clinical course, kinetics of the immune and inflammatory response, as well as pathogen load and shedding [13]. Both microorganisms are frequently isolated from respiratory tract of pigs in the field conditions [1, 27]. A previous study [13], investigating the role of prior SIV infection on the *Hps* colonization, revealed that *Hps* colonization was higher in the nose and lungs of SIV/*Hps* pigs compared to *Hps*-only pigs. These results indicate that SIV infection contributes to enhance bacterial colonization. In SIV/*Hps* pigs IL-8, IL-6 and IL-1β protein levels were increased in the tracheal wash and bronchoalveolar lavage fluid (BALF), and BALF cells IL-8, IL-6 and IL-1β mRNA expression levels were significantly increased over SIV-only and Hps-only pigs.

Based on the results of our study it could be stated that *Hps* did not cause significant lesions in the lung unless pigs were co-infected with SIV. No significant macroscopic lung lesions after experimental infection with *Hps* has been also reported previously [8]. In the same study *Hps* was not isolated from *Hps*-single infected pigs. In our experiment we had been able to isolate of *Hps* in the case of 3 out of 11 *Hps*-single inoculated pigs, but in group co-inoculated with SIV the isolation was successful in 8 out of 11 animals. In the remaining groups (SIV and control) *Hps* was not isolated from lung. Typical systemic lesions of polyserositis were found in only one pig from co-inoculated group. In the present study no significant differences between gross lung lesions has been found between groups single or dually inoculated with SIV but in both groups lung score was significantly higher than in *Hps*-single inoculated group. In addition, more severe clinical signs were observed in dual inoculated pigs compared to single *Hps*- but not SIV-inoculated animals. The more severe clinical course of the infection was probably a consequence of more severe lung lesions present in pigs inoculated with SIV or SIV and *Hps*. Enhanced lung lesions in pigs single or dual inoculated with SIV could be a result of stronger replication of infective agents and more severe local inflammatory responses. Similarly as in the previous study [12] establishing that the titre of Bb was higher in the respiratory tract of SIV/Bb co-infected pigs, we found that SIV enhanced *Hps* colonization of the lung. In accordance to other experiment [19] the significantly higher SIV titre in the nasal swabs and lung was observed in co-inoculated pigs. Simultaneously, no effect of SIV on the *Hps* shedding has been found. These findings suggest that *Hps* can facilitate SIV replication in the respiratory tissue of dual inoculated pigs.

The significant influence of both pathogens on the systemic inflammatory response has been also found in the present study. In the groups inoculated with bacteria (single or dual) the APP response was in general higher than in virus-single inoculated animals. These findings are in accordance with the results of previous studies with various respiratory pathogens of swine [12, 19, 31, 32].

## Conclusions

The results of our study revealed that coinfection with SIV and *Hps* modulates inflammatory responses, clinical course of disease and pathogen load within the respiratory tract in pigs. Co-infection with SIV potentiates the severity of lung lesions and facilitated *Hps* replication in the porcine lung. Furthermore, *Hps* influenced the SIV nasal shedding. Enhanced *Hps* and SIV replication, together with stronger systemic and local inflammatory response contributed to a more severe clinical signs, as well as stronger and earlier immune response in co-inoculated animals compared to single inoculated pigs. Moreover, we confirmed the previous evidence [8] that single-*Hps* infection does not produce significant pneumonic lesions but it should be in mind that other strains of *Hps* may produce lesions different from that reported in the present study.

## Methods

### Virus

The virus used in experiment (avian-like H1N1 A/Poland/Swine/14131/2014 (SIV)), had been isolated from the lung of pig suffering from acute swine influenza. This strain is representative H1N1 SIVs circulating recently in Poland. The stock used for nasal inoculation represented the third passage in eggs. The virus titre was evaluated in Madin-Darby canine kidney (MDCK) cells (ATCC).

### Bacteria

Strain of *Hps*, previously isolated in National Veterinary Research Institute was selected for the experimental infections (isolate PIWetHps192/2015). Strain originating from lung of pig from Polish herd and the analysis of the 16S rRNA gene sequences [33] revealed 99% similarity to *Hps* isolate CN9–2 described by Olvera et al. [34] (classified as moderate virulent serovar 15).

To prepare the inoculum, the strain was streaked onto a pleuropneumonia-like organism (PPLO) agar (Becton Dickinson, USA), supplemented with 10 μg/ml of β-NAD (Sigma-Aldrich, Germany), 1 mg/ml glucose (Avantor Performance Materials, Poland) and 5% horse serum (Sigma-Aldrich, Germany), which was incubated for 24 h at 37 °C in an atmosphere of 8% CO2. Colonies were harvested and suspended in phosphate-buffered saline (PBS) (Life Technologies, USA) to 0.5 McFarland turbidity (which corresponds with approximately 1.5 × 10^8^ colony forming units (CFU)/ml). A plate count was also performed to quantify the accurate number of viable bacteria (final result 1.2 × 10^8^ CFU/ml of *Hps* strain).

### Experimental design

Thirty seven 6-week-old piglets bought from a commercial farm, both sexes, were used in the study (as research animals). Piglets were randomly allocated to 4 groups (*Hps* (*n* = 11); *Hps* + SIV (n = 11); SIV (n = 11); control (*n* = 4) (a sample size calculation based on a resource equation method). The sourced herd was seronegative to P, pseudorabies virus and *Mycoplasma hyopneumoniae*. No evidence of streptococcosis, pleuropneumoniae, Glässers disease and atrophic rhinitis was recorded based on clinical, serological (detection of dermonecrotoxin specific antibodies and antibodies to ApxIV) and pathological examinations (turbinate lesions, polyserositis, polyarthritis, serofibrinous or fibrino-purulent exudate on mucosal surface, arthritis, meningitis). Before the start of the study all piglets were free of influenza A virus and *Hps* antibodies as determined by haemagglutination inhibition assays (HI) using A/Poland/Swine/14131/2014 (H1N1), A/swine/England/96 (H1N2), A/swine/Flanders/1/98 (H3N2), pdm-like H1N1 (A/swine/Poland/031951/12) and Swinecheck® HPS ELISA test (Biovet, Canada).

During the experiment research animals were housed on a (Biosafety Level-3) BSL3 animal facility in four independent units. Animal use and handling protocols were approved by II Local Ethical Commission for the Animal Experiments of University of Life Sciences in Lublin (number of approval: 77/2014).

The animals were acclimatized to BSL3 hygienic conditions for 7 days before commencing the experiment. On day 0, piglets from SIV and *Hps* + SIV groups were inoculated intranasally (IN) with SIV (10^6.8^TCID_50_ in 3 ml of PBS. Piglets from *Hps* and *Hps* + SIV groups were inoculated IN with *Hps* (3.6 × 10^8^ CFU *Hps* in 3 ml of PBS. Four mock-inoculated pigs served as controls.

Pigs were examined daily from day - 7 to 10 post inoculation (DPI) or until euthanasia (at 2 and 4 DPI). Animals were observed and scored for the respiratory signs as follows: respiratory rate: 0 – normal (<34 breaths/min), 1 – slightly elevated (35–40 breaths/min), 2 – moderately elevated (41–45 breaths/min), slight abdominal breathing, 3 – clearly elevated (>46 breaths/min), distinct abdominal breathing; nasal discharge 0 – absent, 1 present; coughing 0 – absent, 1 present; sneezing 0 – absent, 1 present, anorexia 0 – absent, 1 present [17]. Rectal temperature was also measured daily. Hyperthermia was recorded when the rectal temperature reached 40 °C. If the fever was observed 1 additional point was added to the clinical score. All scores per topic were accumulated for a final clinical score calculated for each pig (0–8).

### Sample collection

Nasal swabs were collated daily from all animals (at −7, 0 (inoculation), 1, 2, 3, 4, 5, 6, 7, 8, 9 and 10 DPI). Blood samples were collected at −7, 0 (inoculation), 1, 2, 3, 5, 7 and 10 DPI. Three piglets per inoculated groups were euthanized at 2 and 4 DPI. The remaining inoculated as well as control pigs were euthanized and necropsied at 10 DPI. Complete necropsy was done on each animal, with special emphasis on the respiratory tract. Lung lesions were scored using the method developed by Madec and Kobisch [35] according to the scheme presented in details previously [19]. Samples from lung (apical, middle, caudal right lobes and accessory lobe) were collected for further analyses.

### Laboratory examination

#### Pathogen shedding and load

SIV titration (nasal swabs, lung tissue) in Madin-Darby canine kidney cells (MDCK) (ATCC) were performed in duplicate as described previously [17].

For determination the quantity of *Hps* in samples collected from piglets (nasal swabs and lung tissue fragments) the quantitative real-time PCR was used (all samples were tested in duplicate) [36]. It targets the 392–466 bp region of the inf B gene of *Hps*. Nasal swabs were placed into centrifuge tubes (2 ml), suspended in 1 ml of PBS and after 10 min vortexed for 30 s. The liquid was collected to the new tube (1.5 ml) and suspensions were centrifuged at 3000 *g* during 3 min. The supernatant was discarded and the remaining pellet was resuspended in 100 μl of TRIS buffer (10 mM Tris–HCl pH 8.5) and vortexed for an additional 30 s. Homogenates (50% wt/vol) of middle right lobe (the main site of gross lesions observed in the present study) were prepared in PBS. DNA was extracted from thus prepared samples using the Genomic Mini DNA isolation kit (A&A Biotechnology, Poland) according to the manufacturer instructions. DNA was stored at −70 °C for further analysis.

#### Serum analyses

All sera were examined using HI assays (SIV) against challenge strain and ELISA test against *Hps* as directed by the manufacturer (Swinecheck®HPS, Biovet, Canada). The presence or absence of antibodies to investigated antigen (Ag) was determined by calculating the ELISA ratio. ELISA ratio (Er) was calculated according to the following formula:

$$ \mathrm{Er}=\frac{\mathrm{OD}\ \mathrm{of}\  \mathrm{test}\  \mathrm{serum}\  \mathrm{from}\  \mathrm{wells}\  \mathrm{with}\ \mathrm{Ag}\hbox{-} \mathrm{OD}\ \mathrm{of}\  \mathrm{test}\  \mathrm{serum}\  \mathrm{from}\  \mathrm{wells}\  \mathrm{with}\mathrm{out}\ \mathrm{Ag}}{\mathrm{mean}\ \mathrm{OD}\ \left(+\right)\ \mathrm{from}\  \mathrm{wells}\  \mathrm{with}\ \mathrm{Ag}\hbox{-} \mathrm{mean}\ \mathrm{OD}\ \left(+\right)\ \mathrm{from}\  \mathrm{wells}\  \mathrm{with}\mathrm{out}\ \mathrm{Ag}\ } $$

(+) - positive control

Antibodies against SIVs were measured using a HI assay, performed according to the standard procedure, using 0.5% chicken erythrocytes and 4HA units of challenge strains [37]. All sera were tested in serial twofold dilutions, starting at 1:20. For estimates of the prevalence of antibodies, titres ≥20 were considered positive [38]. For statistical analyses the titres lower than 20 was set as 5.

Acute phase proteins (C-reactive protein (CRP, haptoglobin (Hp), serum amyloid A (SAA) and pig major acute phase protein (Pig-MAP)) were examined using ELISA assays according to producer’s recommendations (Pig C-reactive protein ELISA and Pig haptoglobin ELISA from Life Diagnostics, USA; Pig-MAP KIT ELISA from Acuvet Biotech S.L., Spain; Phase Serum Amyloid A Assay from Tridelta Development Ltd. County Kildare, Ireland). All serum samples were tested in duplicate.

#### Lung proinflammatory cytokine levels

Lung tissues were collected from pigs during necropsy and prepared in PBS (pH 7.4) [12, 16]. 1.0 g of the lung tissue fragments were suspended in 1 ml of PBS, respectively (1:1 *w*/*v*), and frozen before being homogenized. After homogenization, with the use of tissue homogenizer X620 (CAT, Germany), the samples were centrifuged at 3000 *g* for 10 min. The supernatants were collected and stored at −80°C before cytokines analysis was performed using ELISA assay. The ELISA kits specific for porcine cytokines: IL-10, IFN-γ and TNF-α were bought from Invitrogen Corporation (Camarillo, USA), and these used for IL-1β were bought from RayBiotech, Inc. (Norcross, USA). The concentration of IL-6 was determined with the use of IL-6 Pig ELISA Kit from Abcam (Cambridge, UK). All tests were performed in duplicate according to the manufacturers’ recommendations. The quantity of the cytokines was calculated based on standard curve for each cytokine with the use of FindGraph software.

### Statistical analysis

The data were subjected to the W. Shapiro-Wilk’s test of normality and the Levene’a test of equal variances with STATISTICA 8.0 (StatSoft). Differences between means were tested for statistical significance by a nonparametric Kruskal - Wallis test with post hoc multiple comparisons for comparison of all pairs. The Friedman test was used to compare observations repeated on the same subjects. For analysis of correlation the Spearman Rank correlation test was used. For all analyses, *p* < 0.05 was considered statistically significant.

## Abbreviations

Ag: Antigen
APP: Acute phase proteins 19
CFU: Colony forming unit
CRP: C-reactive protein
DPI: Day post infection
Er: ELISA ratio
HI: Haemagglutination inhibition
Hp: Haptoglobin
Hps: *Haemophilus parasuis*
IFN-γ: Interferon gamma
IL: Interleukin
OD: Optical density
PBS: Phosphate buffered saline
Pig-MAP: Pig major acute phase protein
Pm: *Pasteurella multocida*
PRDC: Porcine respiratory disease complex
PRRSV: Porcine reproductive and respiratory syndrome virus
SAA: Serum amyloid A
SIV: Swine influenza virus
TCID50: 50% tissue culture infective dose
TNF-α: Tumor necrosis factor alfa

## References

[1] Fablet C, Marois C, Dorenlor V, Eono F, Eveno E, Jolly JP, Le Devendec L, Kobisch M, Madec F, Rose N (2012). Bacterial pathogens associated with lung lesions in slaughter pigs from 125 herds. Res Vet Sci.

[2] Opriessnig T, Gimenez-Lirola LG, Halbur PG (2011). Polymicrobial respiratory disease in pigs. Anim Health Res Rev.

[3] Choi YK, Goyal SM, Joo HS (2003). Retrospective analysis of etiologic agents associated with respiratory diseases in pigs. Can Vet J.

[4] Truszczyński M, Pejsak Z (2010). Contribution of Pasteurella multocida to the porcine respiratory disease complex. Medycyna Weterynaryjna.

[5] de la Fuente AJ M, Gutierrez Martin CB, Perez Martinez C, Garcia Iglesias MJ, Tejerina F, Rodriguez Ferri EF (2009). Effect of different vaccine formulations on the development of Glasser's disease induced in pigs by experimental Haemophilus parasuis infection. J Comp Pathol.

[6] Simon-Grifé M, Martín-Valls GE, Vilar MJ, Busquets N, Mora-Salvatierra M, Bestebroer TM, Fouchier R, Martín M, Mateu E, Casal J (2012). Swine influenza virus infection dynamics in two pig farms; results of a longitudinal assessment. Vet Res.

[7] Brockmeier SL, Halbur PG, Thacker EL, GJ BKA (2002). Porcine respiratory disease complex. Polymicrobial diseases.

[8] Vahle JL, Haynes JS, Andrews JJ (1995). Experimental reproduction of Haemophilus parasuis infection in swine: clinical, bacteriological, and morphologic findings. J Vet Diagn Investig.

[9] Oliveira S, Pijoan C (2004). Haemophilus parasuis: new trends on diagnosis, epidemiology and control. Vet Microbiol.

[10] Rapp-Gabrielson VJ, Gabrielson DA (1992). Prevalence of Haemophilus parasuis serovars among isolates from swine. Am J Vet Res.

[11] Jablonski A, Zebek S, Kolacz R, Pejsak Z (2011). Usefulness of PCR/RFLP and ERIC PCR techniques for epidemiological study of Haemophilus parasuis infections in pigs. Pol J Vet Sci.

[12] Loving CL, Brockmeier SL, Vincent AL, Palmer MV, Sacco RE, Nicholson TL (2010). Influenza virus coinfection with Bordetella bronchiseptica enhances bacterial colonization and host responses exacerbating pulmonary lesions. Microb Pathog.

[13] Loving C, Brockmeier S, Vincent A. Prior swine influenza virus infection enhances pulmonary responses to secondary Haemophilus parasuis infection. In: Proceedings of the Pig Veterinary Society International Congress. Perry: IPVS; 2010. p. 264.

[14] Oliveira S, Galina L, Blanco I, Canals A, Pijoan C (2003). Naturally-farrowed, artificially-reared pigs as an alternative model for experimental infection by Haemophilus parasuis. Can J Vet Res.

[15] Barigazzi G, Valenza F, Bollo E. Anatomohistopathological features related to Haemophilus parasuis infection in pigs. In: Proceedings of the Pig Veterinary Society International Congress. IPVS: Bangkok; 1994. p. 235.

[16] Pomorska-Mol M, Markowska-Daniel I, Kwit K, Czyzewska E, Dors A, Rachubik J, Pejsak Z (2014). Immune and inflammatory response in pigs during acute influenza caused by H1N1 swine influenza virus. Arch Virol.

[17] Pomorska-Mol M, Kwit K, Markowska-Daniel I, Kowalski C, Pejsak Z (2014). Local and systemic immune response in pigs during subclinical and clinical swine influenza infection. Res Vet Sci.

[18] Pomorska-Mol M, Markowska-Daniel I, Kwit K, Stepniewska K, Pejsak Z (2015). Profile of the porcine acute-phase proteins response following experimental co-infection with H3N2 swine influenza virus and Pasteurella multocida. Biomarkers.

[19] Pomorska-Mól M, Dors A, Kwit K, Kowalczyk A, Stasiak E, Pejsak Z (2017). Kinetics of single and dual infection of pigs with swine influenza virus and Actinobacillus pleuropneumoniae. Vet Microbiol.

[20] Pomorska-Mól M, Markowska-Daniel I, Kwit K, Urbaniak K, Pejsak Z. Correlation between serum acute phase proteins, lung pathology and disease in pigs after H3N2 swine influenza virus and Bordetella bronchiseptica experimental co-infection. BULL VET INST PULAWY 2015;59:1-7.

[21] Brown IH (2000). The epidemiology and evolution of influenza viruses in pigs. Vet Microbiol.

[22] van Reeth K, Nauwynck H (2000). Proinflammatory cytokines and viral respiratory disease in pigs. Vet Res.

[23] Avadhanula V, Rodriguez CA, Devincenzo JP, Wang Y, Webby RJ, Ulett GC, Adderson EE (2006). Respiratory viruses augment the adhesion of bacterial pathogens to respiratory epithelium in a viral species- and cell type-dependent manner. J Virol.

[24] Astry CL, Jakab GJ (1984). Influenza virus-induced immune complexes suppress alveolar macrophage phagocytosis. J Virol.

[25] Didierlaurent A, Goulding J, Patel S, Snelgrove R, Low L, Bebien M, Lawrence T, van Rijt LS, Lambrecht BN, Sirard JC (2008). Sustained desensitization to bacterial toll-like receptor ligands after resolution of respiratory influenza infection. J Exp Med.

[26] Azoulay-Dupuis E, Lambre CR, Soler P, Moreau J, Thibon M (1984). Lung alterations in guinea-pigs infected with influenza virus. J Comp Pathol.

[27] Fablet C, Marois-Crehan C, Simon G, Grasland B, Jestin A, Kobisch M, Madec F, Rose N (2012). Infectious agents associated with respiratory diseases in 125 farrow-to-finish pig herds: a cross-sectional study. Vet Microbiol.

[28] Pomorska-Mol M, Markowska-Daniel I, Kwit K, Stepniewska K, Pejsak Z (2013). C-reactive protein, haptoglobin, serum amyloid a and pig major acute phase protein response in pigs simultaneously infected with H1N1 swine influenza virus and Pasteurella multocida. BMC Vet Res.

[29] Pomorska-Mol M, Markowska-Daniel I, Kwit K (2012). Acute phase protein response in pigs experimentally co-infected with swine influenza virus and Bordetella bronchiseptica. Cen Eur J Immunol.

[30] Brockmeier SL, Loving CL, Nicholson TL, Palmer MV (2008). Coinfection of pigs with porcine respiratory coronavirus and Bordetella bronchiseptica. Vet Microbiol.

[31] Thacker EL, Thacker BJ, Janke BH (2001). Interaction between Mycoplasma hyopneumoniae and swine influenza virus. J Clin Microbiol.

[32] Thacker EL, Halbur PG, Ross RF, Thanawongnuwech R, Thacker BJ (1999). Mycoplasma hyopneumoniae potentiation of porcine reproductive and respiratory syndrome virus-induced pneumonia. J Clin Microbiol.

[33] Olvera A, Calsamiglia M, Aragon V (2006). Genotypic diversity of Haemophilus parasuis field strains. Appl Environ Microbiol.

[34] Olvera A, Cerdà-Cuéllar M, Nofrarías M, Revilla E, Segalés J, Aragon A: Dynamics of Haemophilus parasuis genotypes in a farm recovered from an outbreak of Glässer's disease. 2007, 123(1–3):230–237. 10.1016/j.vetmic.2007.03.004 17418506

[35] Madec F, Kobisch M. Bilan lésionnel des porcs charcutiers à l’abattoir. Journées de la Recherche Porcine en France. 1982;14:405–12.

[36] Turni C, Pyke M, Blackall PJ (2017). Validation of a real-time PCR for Haemophilus parasuis. J Appl Microbiol.

[37] Pomorska-Mol M, Markowska-Daniel I, Pejsak Z (2012). Acute phase protein response during subclinical infection of pigs with H1N1 swine influenza virus. Vet Microbiol.

[38] Pomorska-Mol M, Czyzewska-Dors E, Kwit K, Wierzchoslawski K, Pejsak Z. Ceftiofur hydrochloride affects the humoral and cellular immune response in pigs after vaccination against swine influenza and pseudorabies. BMC Vet Res. 2015;11:268. 10.1186/s12917-015-0586-3 PMC4618681 26493336

